# Recent Advances in Gels for Water Treatment

**DOI:** 10.3390/gels11070519

**Published:** 2025-07-03

**Authors:** Anita-Laura Chiriac, Andrei Sarbu, Anamaria Zaharia

**Affiliations:** Advanced Polymer Materials and Polymer Recycling Group, National Institute for Research & Development in Chemistry and Petrochemistry (ICECHIM), 060021 Bucharest, Romania; anita-laura.radu@icechim.ro (A.-L.C.); andrei.sarbu@icechim.ro (A.S.)

## 1. Introduction

Water is a vital resource for all known life forms on our planet, and essential for human health, economic development, and maintaining ecological balance. It is involved in many biological, ecological, and geochemical processes, and access to quality water is a fundamental indicator of environmental sustainability [1]. However, this indispensable resource is increasingly exposed to risks generated by a wide range of contaminants, many of which originate from anthropogenic activities such as industrial discharges, agricultural runoff, inadequate management of household waste, and emissions from transport systems. These activities contaminate water bodies with emerging pollutants, including organic and inorganic substances such as heavy metals, pesticides, pharmaceuticals, personal care products, industrial substances, and micro- and nanoplastics. Emerging contaminants often require specific remediation methods adapted to their unique chemical properties, which makes their removal from water more difficult. As the complexity of contaminants in the aquatic environment increases, traditional remediation methods are becoming ineffective in removing substances such as endocrine disruptors or microplastics. These challenges highlight the need for advanced remediation technologies to treat a broader spectrum of pollutants effectively [2,3].

Although effective in many contexts, conventional water treatment technologies, including coagulation–flocculation, activated carbon adsorption, and membrane filtration, often face limitations, such as low selectivity, high operational costs, membrane contamination, and incomplete degradation of pollutants when attempting to remove emerging contaminants efficiently [4]. As a result, there has been a growing interest in developing innovative materials and advanced plating methods that offer superior performance in improved selectivity, reusability, and environmental compatibility.

Among the innovative materials under intense investigation, gel-based systems have emerged as promising candidates due to their unique physicochemical properties. Hydrogels, aerogels, oleogels, composite gels, and nano-hybrids are characterized by high porosity, tunable surface chemistry, ability to incorporate functional groups or nanoparticles, versatility, and reusability, as well as environmental compatibility, thus facilitating their adaptation to a wide range of water purification applications [5]. These innovative materials can be designed to adsorb or degrade specific contaminants selectively, serve as platforms for catalytic or photocatalytic reactions, or act as real-time water quality monitoring sensors.

Scientific research in this area is evolving rapidly, with remarkable progress in synthesizing, characterizing, and functionalizing gels for water treatment. The applications of these materials extend beyond conventional purification processes, addressing complex topics such as industrial wastewater remediation, selective retention of trace-level pollutants [6], controlled release of remediation agents [7], and their use in environmental sensing and biocatalysis processes.

This Special Issue of *Gels*, entitled “Gels for Water Treatment”, aims to bring together original research and recent synthesis papers aimed at providing innovative insights and enriching knowledge on the latest approaches in the development of gels for water treatment. This Special Issue includes ten articles that explore sustainable applications for water treatment, as well as the development of advanced and innovative materials, including articles that examine existing methods. Of these, seven articles cover a wide range of topics, including the development of novel hydrophobic cryogel structures based on amino acids, magnetic polymer gels made from chitosan–Fe, highly porous hybrid aerogels derived from chitosan–silica, dual-responsive hydrogels, biocomposite hydrogels based on tragacanth gum with activated carbon, and a new green coagulant in the form of a gel. This issue also includes three review articles, covering various aspects of fundamental research and practical applications involving new generations of gels designed for water treatment.

## 2. Overview of the Papers Published in This Special Issue

This Special Issue, “Gels for Water Treatment,” brings together seven research articles and three review papers highlighting recent advancements in gel-based wastewater treatment technologies. These contributions explore innovative fabrication techniques, the properties of new gels, and their potential for industrial and large-scale applications. A brief introduction to the included articles is given below.

The paper “Amino Acid-Based Hydrophobic Cryogels for Efficient Methylene Blue Removal: A Reusable and Eco-Friendly Approach to Dye-Contaminated Wastewater Treatment” by Sofuoğlu et al. reports the synthesis of a novel hydrophobic cryogel for the efficient removal of methylene blue dye from water. The cryogel demonstrated a high adsorption capacity (1304.6 mg/g), rapid uptake (within 30 min), and strong reusability (up to 98% efficiency over five cycles). Adsorption followed a monolayer chemisorption model, driven by hydrophobic interactions, and exhibited improved performance at higher temperatures and salt concentrations. The cryogel also performed well in real textile wastewater, highlighting its practical potential as a sustainable, high-performance adsorbent for the treatment of dye-contaminated water.

The second paper, “Development of Chitosan Polysaccharide-Based Magnetic Gel for Direct Red 83:1 Removal from Water” by Murcia-Salvador et al., presents the synthesis and evaluation of a chitosan–Fe magnetic polymeric gel for removing the azo dye Direct Red 83:1 from water. Under optimal conditions (1 g, pH 7), the gel showed a maximum adsorption capacity of 17.46 mg/g. The process followed pseudo-second-order kinetics and fit the Temkin isotherm, indicating heterogeneous surface adsorption. The gel’s magnetic properties enabled easy separation, making it an efficient and practical alternative for treating dye-contaminated water, particularly in wastewater from the dyeing industry.

The paper entitled “Denaturing Gradient Gel Electrophoresis Approach for Microbial Shift Analysis in Thermophilic and Mesophilic Anaerobic Digestions” uses PCR-DGGE (denaturing gradient gel electrophoresis) to analyze microbial community shifts during the anaerobic digestion of dairy manure under different temperatures (28–52 °C). The results showed that temperature and incubation time significantly influenced microbial composition, with distinct communities forming under mesophilic and thermophilic conditions. Initially dominated by *Acinetobacter* sp., the microbial populations evolved over 60 days, with thermophilic reactors favoring *Coprothermobacter proteolyticus*, while mesophilic conditions supported bacteria like *Sphaerochaeta* and *Galbibacter mesophilus*. These microbial shifts are linked to greenhouse gas emissions, particularly methane, highlighting the importance of understanding microbial dynamics for enhancing waste treatment and mitigating emissions. The study demonstrates that DGGE is a valuable tool for tracking microbial changes in anaerobic systems.

The paper “Chitosan–Silica Composite Aerogel for the Adsorption of Cupric Ions” by Varda et al. describes a lightweight, highly porous chitosan–silica hybrid aerogel synthesized for removing copper (II) ions from contaminated water. Made using chitosan from fishery waste and silica, the aerogel features a 96% porosity, a bulk density of 56 kg/m^3^, and a macroporous structure with some micro- and mesoporosity. It demonstrated a maximum adsorption capacity of ~40 mg/g and reached equilibrium in less than an hour, with surface reactions being the rate-limiting step. The material’s performance was primarily unaffected by pH and benefited from functional groups, such as –NH_2_ and –OH. This low-cost, eco-friendly adsorbent demonstrates strong potential for sustainable water treatment applications.

The paper entitled “Dual-Responsive Hydrogels for Mercury Ion Detection and Removal from Wastewater” proposes photopolymerized hydrogels incorporating chelating agents and a rhodamine B derivative for the simultaneous detection and removal of Hg^2+^ ions from water. Among the five hydrogel types synthesized, those containing phytic acid showed the highest mercury adsorption capacity (up to 56 mg/g) and enhanced mechanical strength. The findings demonstrate a fast, cost-effective, and dual-function approach for mercury remediation and monitoring in wastewater.

The paper entitled “Activated Carbon-Incorporated Tragacanth Gum Hydrogel Biocomposite: A Promising Adsorbent for Crystal Violet Dye Removal from Aqueous Solutions” by Thamer et al. presents the development of a biocomposite hydrogel formed by thermally cross-linking tragacanth gum with activated carbon derived from pomegranate peels. This hydrogel was evaluated as an adsorbent for removing the cationic dye crystal violet from aqueous solutions. The material was shown to possess high adsorption capacity for cationic dyes, highlighting its potential as an efficient and environmentally friendly option for water treatment.

The paper “Assessment of Prickly Pear Fruit Peel Mucilage in Form of Gel as a Green Coagulant for the Tertiary Treatment of Domestic Wastewater” by Otálora et al. evaluates the use of prickly pear fruit peel mucilage gel as a green biodegradable coagulant for the tertiary treatment of domestic wastewater. The mechanisms of floc formation differed: mucilage worked via adsorption/bridging, while FeCl_3_ relied on charge neutralization. The mucilage also captured a higher amount of organic matter in the flocs. Despite its promising environmental benefits, the large-scale application of this biocoagulant faces challenges related to production cost, market integration, and regulatory approval. Future research should explore its efficacy in treating high-pH industrial effluents, such as those from the leather and textile industries.

The review entitled “New Trends in Preparation and Use of Hydrogels for Water Treatment” by Sandu et al. highlights recent advancements in polymer-based hydrogels for wastewater treatment, emphasizing their high efficiency, sustainability, reusability, and ability to target specific contaminants. Compared to traditional methods, hydrogels offer advantages such as low energy consumption, flexibility, and environmental friendliness. Innovations in synthesis and hybrid materials have improved adsorption performance without compromising biodegradability. However, scalability, long-term stability, functionalization, regeneration efficiency, and environmental impact remain key considerations. The review compares hydrogel types based on their synthesis methods, raw materials (synthetic, natural, or hybrid), target pollutants (e.g., dyes, heavy metals), and key properties, including adsorption kinetics, capacity, and reusability.

The review “Radiation-Induced Hydrogel for Water Treatment” by Haque et al. explores various hydrogels synthesized using different radiation sources, particularly gamma and electron beam radiation from Co-60 and electron guns. It discusses optimal radiation doses, suitable monomer and polymer compositions, and their effectiveness in water purification. The authors also compare radiation methods (e.g., UV–visible, X-ray, microwave) and highlight the advantages of specific techniques while outlining current trends and prospects in radiation-induced hydrogel development.

The review entitled “Natural Polysaccharide-Based Hydrogels Used for Dye Removal” by Stanciu et al. focuses on the past decade’s advancements in using these biodegradable, biocompatible, and sustainable materials for dye removal. It covers synthesis methods, adsorption performance, influencing factors, kinetic and isotherm models, thermodynamic data, recyclability, and future research directions.

## 3. Conclusions and Outlook

Gels offer a versatile and environmentally friendly platform for advanced water treatment. With ongoing innovations in material design, functionalization, and process integration, gels will become a key component of next-generation water purification technologies. Continued interdisciplinary research and collaboration with industry will be crucial for translating lab-scale successes into practical, real-world solutions.

The editors sincerely acknowledge the authors’ valuable contributions to this field and hope that their work will garner growing recognition and impact in the future.
